# The contributions of resting-state functional-MRI studies to our understanding of male patients with obstructive sleep apnea: a systematic review

**DOI:** 10.3389/fneur.2025.1532037

**Published:** 2025-04-09

**Authors:** Ruoxi Yu, Yan Li, Kangqing Zhao, Fangfang Fan

**Affiliations:** ^1^National Institute of Traditional Chinese Medicine Constitution and Preventive Medicine, Beijing University of Chinese Medicine, Beijing, China; ^2^Hangzhou MindMatrixes Technology Co., Ltd, Hangzhou, China; ^3^Peking University Sixth Hospital, Peking University Institute of Mental Health, NHC Key Laboratory of Mental Health (Peking University), National Clinical Research Center for Mental Disorders (Peking University Sixth Hospital), Beijing, China; ^4^Department of Neurology, Beth Israel Deaconess Medical Center and Harvard Medical School, Boston, MA, United States

**Keywords:** resting-state fMRI, obstructive sleep apnea, functional connectivity, functional segregation, functional integration

## Abstract

**Objectives:**

Obstructive sleep apnea (OSA) is a condition marked by the recurrent partial or complete obstruction of the upper airway during sleep. This leads to intermittent pauses in breathing, fragmented sleep, and frequent awakenings throughout the night. Many of these symptoms are believed to be linked to brain damage; however, the fundamental neurological processes underlying these impairments remain largely unknown. This study investigates resting-state functional MRI (rs-fMRI) findings in male patients with OSA to better understand the specific mechanisms associated with this condition in this demographic.

**Methods:**

The search was conducted in the PubMed and Google Scholar databases, encompassing literature from their inception to July 2024. Studies were identified based on predetermined inclusion and exclusion criteria and were evaluated by two independent reviewers.

**Results:**

A total of 16 eligible original rs-fMRI studies on male patients with OSA were included in this review. The findings indicate that patients with OSA exhibit alterations in resting-state brain activity. These neural changes may help explain the effects of OSA on emotion, cognition, and quality of life. Additionally, these findings could be used in the future to evaluate treatment outcomes.

**Conclusion:**

This study highlights significant changes in local brain activities, interested region related functional connectivity, and whole-brain functional connectivity networks in patients with OSA. These findings offer valuable insights into the neural alterations at the core of OSA and may serve as potential biomarkers for diagnosis and intervention.

## 1 Introduction

Obstructive sleep apnea (OSA) is a condition characterized by recurrent episodes of partial or complete collapse of the upper airway during sleep, resulting in intermittent apnea, sleep fragmentation, and associated cortical arousal or a drop in blood oxygen saturation (1, 2). Furthermore, OSA is linked to a range of health complications, including high blood pressure, stroke, heart disease, and various types of cancer. The heightened inflammation occurring at both local and systemic levels due to OSA is believed to be one of the underlying biological processes connecting this sleep disorder to the aforementioned health issues (3, 4).

The prevalence of OSA in young adults (18–30 years) is approximately 16%, with a higher prevalence in middle-aged and older populations (5). Risk factors for OSA include male sex, obesity, age between 40 and 70 years, and familial aggregation (6, 7). Suspected risk factors include genetics, smoking, menopause, alcohol use before bedtime, and nocturnal nasal congestion (8). Among these, obesity stands out as the primary risk factor for developing OSA. Studies have indicated that over 40% of individuals with a body mass index (BMI) exceeding 30 exhibit OSA, with this percentage rising to 60% among those diagnosed with metabolic syndrome (9). Male sex is another significant risk factor, although the biological basis for sex differences in OSA remains unclear (10). OSA is more commonly observed in men than in women. Studies suggest that the condition affects approximately 3% of women and 12% of men aged 30–49 years, and 8% of women and 18% of men aged 50–70 years (11). Thus, conducting research on OSA in men is crucial for understanding the specific risks and mechanisms associated with the condition in this demographic. This understanding can lead to more effective prevention strategies, early diagnosis, and targeted treatments, ultimately improving the quality of life and health outcomes for affected individuals.

Over the past 20 years, a variety of methods have been extensively employed to study the neurophysiological aspects of OSA, including functional magnetic resonance imaging (fMRI) (12), electroencephalography (EEG) (13), positron emission tomography (14), and functional near-infrared spectroscopy (15). Among these, EEG-based polysomnography (PSG) is recognized as the gold standard for diagnosing sleep disorders in clinical practice (16, 17). Notably, EEG and fMRI have demonstrated the ability to collect and analyze data with greater efficiency, suggesting they may offer enhanced utility in the clinical diagnosis of various diseases.

In the past decade, resting-state fMRI (rs-fMRI) has become a widely used tool in sleep disorder research, significantly advancing our understanding of the pathophysiology and potential compensatory mechanisms of conditions like obstructive sleep apnea (OSA) (18). rs-fMRI offers a non-invasive and effective method for exploring brain activity (19). With the development of various analytical techniques for rs-fMRI, its application in OSA studies continues to expand, promising further insights into the mechanisms underlying the disease (20). In rs-fMRI studies of OSA, analytical methods can be broadly categorized into two types: local features and functional connectivity (FC) (12). Local features primarily include amplitude of low-frequency fluctuation (ALFF) and regional homogeneity (ReHo). Previous studies have found significant changes in ALFF and ReHo in multiple brain regions of OSA patients. For example, OSA patients exhibit reduced ALFF in the precuneus, posterior cingulate cortex (PCC), angular gyrus (AG), and inferior parietal lobule (IPL), while increased ALFF has been observed in the posterior cerebellum, fusiform gyrus, and frontal lobe (21, 22). The analysis of FC mainly focuses on the default mode network (DMN), salience network (SN), and central executive network (CEN), with DMN dysfunction being the most commonly reported finding in rsfMRI studies of OSA (12). Previous studies have documented abnormalities in the internal connectivity of the DMN, alterations in its global and local characteristics, as well as dysregulation of its structure. In addition to DMN, functional connectivity abnormalities in CEN and SN have also been reported in OSA patients (22, 23). One of the most notable changes is the altered FC between the prefrontal cortex (PFC) and the insula. For instance, Zhang et al. found reduced internal FC in the dorsolateral prefrontal cortex (DLPFC) of OSA patients, while Yu et al. reported decreased FC between the right dorsal amygdala (DA) and the right PFC (23, 24).

Given the high prevalence of OSA and its severe impact on health, the specific risks faced by the male population, the potential applications of rs-fMRI in OSA research, and the urgent need for future research directions, systematically summarizing and analyzing the progress of rs-fMRI studies in male OSA patients is crucial for understanding the specific risks and related mechanisms in this population. Through these studies, we aim to identify brain alterations, providing insights into the pathophysiological changes underlying certain clinical manifestations and offering a scientific basis for early screening, precise diagnosis, and personalized interventions for OSA. This review examines the research progress of rs-fMRI in the study of male patients with OSA and makes suggestions for further research. First, we review various types of rs-fMRI analytical techniques utilized in OSA research. Second, we outline the main modality-specific findings from rs-fMRI studies in male patients with OSA. Finally, we explore the current state of OSA research and propose future directions, including examining the comorbidities associated with OSA and considering the potential of innovative methodologies such as combined EEG-fMRI, machine learning algorithms, and the study of comorbid conditions.

## 2 Materials and methods

We conducted a systematic review of rs-fMRI studies in male patients with OSA, following the PRISMA (Preferred Reporting Items for Systematic Reviews and Meta-Analyses) 2020 guidelines.

### 2.1 Search strategy

The search was performed in the PubMed and Google Scholar databases, covering literature from their establishment to July 2024. To systematically investigate the application of rs-fMRI in male patients with OSA, we searched the title, abstract, and keywords of relevant studies. The search terms included: “obstructive sleep apnea” or “OSA” or “sleep-related breathing disorders” or “sleep apnea” and “resting-state fMRI” or “functional connectivity” or “functional network connectivity” or “functional disconnection.” Papers written in languages other than English were excluded. Additionally, the references of selected articles were screened for further relevant records.

### 2.2 Eligibility criteria

Published original articles were included based on the following inclusion criteria:

1. Articles written in English.
2. Male patients diagnosed with OSA.
3. Patients aged 18 years or older.
4. Studies including male patients with OSA and healthy controls.
5. Use of rs-fMRI to analyze its relationship with OSA.

Exclusion criteria were as follows:

1. Non-population-based studies.
2. Papers unrelated to the research questions upon review.
3. Abstracts, letters, reviews, or other non-research articles.
4. Studies lacking sufficient data on the participants’ lesion characteristics.
5. Studies including female OSA patients and male patients were not separately analyzed.

The titles and abstracts of studies were independently screened by two authors according to the inclusion and exclusion criteria. Discrepancies were resolved through discussion.

### 2.3 Selection process

**Figure 1:**
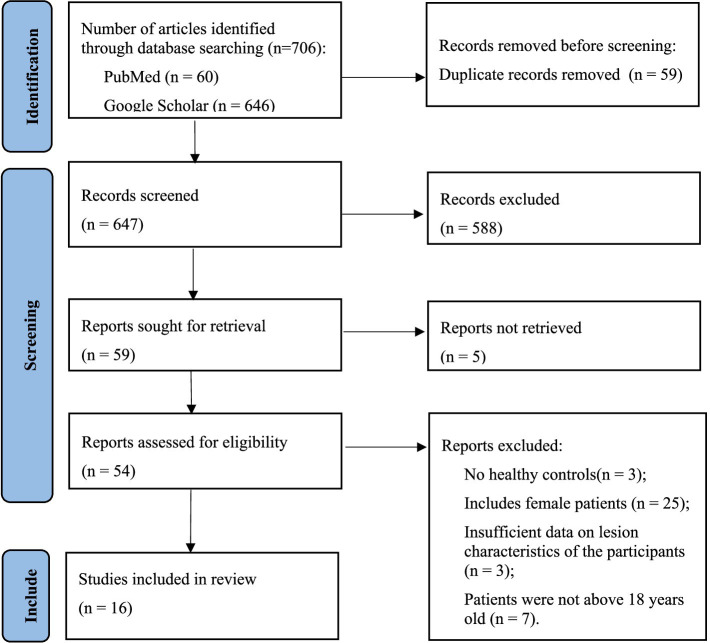
Flow diagram for literature search.

All investigators received professional training in evidence-based medicine, imaging, and computer science and technology to conduct this systematic review. After excluding duplicate articles and uploading potentially eligible studies into Endnote X9 software, two reviewers independently screened the titles, abstracts, and keywords of all retrieved records and reviewed the full text of the articles. Based on the aforementioned inclusion and exclusion criteria, the retrieved studies were screened. Any discrepancies between the two reviewers were resolved through discussion, with a third party assisting in making the final decision. The PRISMA flowchart illustrating the study selection process is shown in Figure 1.

### 2.4 Quality assessment

Two researchers will analyze the literatures using the Risk of Bias in Non-randomized Studies of Exposures (ROBINS-E) tool. ROBINS-E consists of seven assessment domains: (1) risk of bias due to confounding, (2) risk of bias arising from measurement of the exposure, (3) risk of bias in selection of participants into the study, (4) risk of bias due to post-exposure interventions, (5) risk of bias due to missing data, (6) risk of bias arising from measurement of outcomes, (7) risk of bias in selection of the reported result. Each assessment domain contains a series of questions with response categories: “yes,” “probably yes,” “probably no,” “no,” and “no information.” Ultimately, the risk level for each domain will be comprehensively evaluated and classified as “low risk,” “some concerns,” “high risk” or “very high risk.” The risk levels for all seven domains will be obtained.

### 2.5 Data items

The following details were extracted from each study:

1. Characteristics of the papers (authors and publication year).
2. Characteristics of the population (number of participants, inclusion of controls, apnea–hypopnea index of patients, and mean age of all participants).
3. Study design and measurement methods.
4. Methods of analysis.
5. Results/Findings.

Two authors independently performed data extraction. Any discrepancies were resolved through discussion.

### 2.6 Synthesis methods

We employed a narrative synthesis approach to integrate and summarize the study findings. This review follows the narrative synthesis framework proposed by Popay et al. (25), which includes: (1) Organization and Classification of Results – summarizing the main characteristics, objectives, and findings of the included studies and categorizing them into different themes; (2) Pattern Recognition Across Studies – comparing findings from different studies to identify consistencies and discrepancies; (3) Theoretical Interpretation – exploring potential mechanisms and reasons for variations among studies based on existing theoretical frameworks; and (4) Assessment of Limitations – analyzing the quality of the included studies, risk of bias, and the limitations inherent in the narrative synthesis approach itself.

## 3 Results

A total of 706 studies were retrieved from the database. After removing duplicates, 647 studies remained. We then excluded studies based on the title, abstract, and keywords, as they were not related to the main issues discussed in this systematic review (*n* = 588). Of the remaining 59 studies, 5 were excluded because they could not be retrieved. From the remaining 54 studies, 3 involved patients younger than 18 years, 25 involved female patients, 3 did not include healthy controls, and 3 others lacked sufficient pathological characteristics of the participants. Finally, a total of 16 eligible original studies were included in this review. The flowchart of the literature screening and qualification review is shown in Figure 1.

### 3.1 Study quality assessment

In this review, two of the four authors independently assessed the quality of all included studies using the ROBINS-E risk of bias assessment tool. Nine studies were judged as moderate risk of bias and 7 studies were at low risk of bias. Following discussions among the four researchers, the evaluation results are presented in Table 1.

**Table 1:** ROBinS-E risk of bias assessment.

Study	Confounding	Exposure	Selection	Post-exposure	Missing data	Measurement of outcomes	Reported result	Overall bias
Zhang et al. (2013) (23)	Low	Some concerns	Low	Low	Low	Low	Low	Some concerns
Peng et al. (2014) (22)	Low	Some concerns	Low	Low	Low	Low	Low	Some concerns
Li et al. (2015) (47)	Low	Some concerns	Low	Low	Low	Low	Low	Some concerns
Zhang et al. (2015) (49)	Low	Low	Some concerns	Low	Low	Low	Low	Some concerns
Li et al. (2016) (57)	Low	Low	Low	Low	Low	Low	Low	Low
Li et al. (2016) (50)	Low	Low	Low	Low	Low	Low	Low	Low
Chen et al. (2017) (55)	Low	Low	Some concerns	Low	Low	Low	Low	Some concerns
Chen et al. (2018) (56)	Low	Low	Low	Low	Low	Low	Low	Low
Chen et al. (2018) (24)	Low	Low	Some concerns	Low	Low	Low	Low	Some concerns
Yu et al. (2019) (51)	Low	Low	Low	Low	Low	Low	Low	Low
Kang et al. (2020) (48)	Low	Low	Some concerns	Low	Low	Low	Low	Some concerns
Qin et al. (2020) (52)	Low	Low	Some concerns	Low	Low	Low	Low	Some concerns
Kong et al. (2022) (53)	Low	Low	Low	Low	Low	Low	Low	Low
Liu et al. (2022) (54)	Low	Low	Low	Low	Low	Low	Low	Low
Zeng et al. (2022) (21)	Low	Low	Some concerns	Low	Low	Low	Low	Some concerns
Li et al. (2024) (20)	Low	Low	Low	Low	Low	Low	Low	Low

Confounding: risk of bias due to confounding; Exposure: risk of bias arising from measurement of the exposure; Selection: risk of bias in selection of participants into the study; Post-exposure: risk of bias due to post-exposure interventions; Missing data: risk of bias due to missing data; Measurement of outcomes: risk of bias arising from measurement of outcomes; Reported result: risk of bias in selection of the reported result.

### 3.2 rs-fMRI in OSA

rs-fMRI does not require complex task designs, making it easy to operate. It avoids the incomparability of experimental results caused by variations in task designs or the performance of the subjects. rs-fMRI explores spontaneous neural activity and functional connectivity within the brain by measuring the brain’s BOLD signal at rest (26). Since the mid-1990s, this technique has become an important tool in neuroscience research, particularly for exploring functional networks in the brain (27).

When analyzing rs-fMRI data, it is possible to extract information about the function of specific brain regions or the functional connectivity between different brain regions. Analytic approaches can be broadly divided into two types: functional segregation and functional integration (27). Functional segregation includes two commonly used methods: amplitude of low-frequency fluctuations (ALFF) (28, 29) and regional homogeneity (ReHo) (30), while functional integration includes three commonly used methods: seed-based analysis (31), independent component analysis (ICA) (32), and graph theory (33).

Functional segregation focuses on the distinct roles performed by specific brain regions and is primarily used to create detailed brain maps. In contrast, functional integration is concerned with understanding the interactions and connections between disparate brain regions, treating the brain as a cohesive network. Techniques for functional segregation rely on analyzing localized activity within the rs-fMRI data, while those for functional integration focus on examining the connectivity patterns evident in the rs-fMRI data (31).

Most of the previous rs-fMRI studies on OSA focused on functional segregation. Both ALFF and ReHo methods can be used to reveal local neural activity in the brain (18, 34).

ALFF focuses on the intensity of low-frequency fluctuations within a local region and is used to assess the intensity of spontaneous brain activity. Regions with higher ALFF values indicate stronger neural activity in these regions (35). The ALFF method measures the total power of the BOLD signal within the low-frequency range of 0.01 to 0.08 Hz (36). The ALFF method offers the advantage of simplicity, as it does not require any underlying hypothesis. Additionally, ALFF demonstrates high temporal stability and strong test–retest reliability over long periods, typically around 6 months (29). Increasingly, research utilizes ALFF as an index to investigate the intrinsic brain activity traits of OSA and its potential mechanisms (37).

ReHo measures the synchrony of the time series of a voxel with its surrounding voxels, reflecting the consistency of neural activity within a local region. A higher ReHo value indicates better consistency among local voxels and more coordinated neural activity within the local area, though it does not necessarily imply that the local neural activity is more significant (38). ReHo is usually calculated within a low-frequency range, typically between 0.01 Hz and 0.1 Hz, and can be further divided into different frequency bands. ReHo in the lower frequency range (0.01–0.04 Hz) is particularly sensitive to cortical activity (35). The simplicity of the calculations and the reliable characterization make ReHo a potentially useful tool for analyzing resting-state functional MRI data. ReHo could be regarded as a measure for investigating whether local synchronization of spontaneous brain activity is associated with OSA traits (39). Using ReHo, a significant pattern of abnormal local cortical and subcortical connectivity has been observed in patients with OSA (18).

In ReHo and ALFF analyses, overlapping regions indicate areas that are not only active within the same time-frequency domain but also exhibit synchronous activity with neighboring voxels (35). This overlap suggests that these regions are active and involve a relatively large population of neurons. Therefore, although the two indicators point to different aspects, using them together in the analysis process can provide better interpretive power.

Functional integration, a widely utilized approach in rs-fMRI, examines the statistical correlations between signals across different brain regions.

In the application to OSA, seed-based analysis is a commonly used fMRI technique that studies functional connections (FC) between these seed points and other regions of the brain by selecting specific brain regions as seed points. This approach helps to identify abnormal patterns of functional connectivity in the brains of OSA patients, thereby revealing the cognitive impairments and neuropathological mechanisms associated with OSA. First, a brain region or multiple brain regions (each voxel within these regions must first maintain a high degree of consistency in the time series) are identified as the regions of interest (ROIs). Then, the average time series within the ROI is extracted, and the Pearson correlation coefficient is calculated between the ROIs or between each ROI and the time series of all brain voxels, thereby yielding a seed-based FC map (40). Seed-based analysis necessitates the pre-specification of seeds, typically selected based on hypotheses or prior research findings. These seeds can also be identified from metrics such as ALFF or ReHo (41). A primary benefit of this approach is its computational simplicity and the straightforward interpretation of results. The correlation in activation across various brain regions suggests that they are involved in the same underlying functional process, which is interpreted as indicative of functional connectivity (42). Utilizing this method, the brain’s overall connectivity pattern can be graphically represented through a connectivity matrix that illustrates the strength of connections across all seed regions. This type of matrix is frequently utilized in clinical settings. The extensive clinical applications of rs-fMRI have been well-documented in prior reviews (19).

In studies of OSA patients, ICA has been used to analyze fMRI data, revealing alterations in large-scale brain networks. ICA is a model-free technique that separates BOLD signals into independent functional networks using multivariate decomposition (43). Each network corresponds to synchronized neuronal groups, identified through spatial graphs of z-scores (44). The *z*-scores reflect the correlation between voxel time-series and the network’s average time-series, with the mean *z*-score indicating the strength of functional connectivity within the network. This method enables the identification of distinct brain networks without predefined assumptions (41). ICA offers several advantages over seed-based methods, primarily because it requires fewer assumptions about the data beforehand. However, it does necessitate that the user manually identify significant components and differentiate between noise and actual physiological signals (27).

Graph theory has been widely used to examine complex networks, including in OSA research, where it serves as a powerful tool for describing whole-brain networks (18). In graph analysis, nodes can represent voxels, regions of interest (ROIs), or even entire brain networks, and the connections between them, referred to as edges, reflect their functional connectivity. The graph’s connectional characteristics can then be quantified using various parameters. These analyses offer insights into the organizational structure of brain networks in OSA (45). The following are some of the key graph analysis parameters (46):

1. Clustering coefficient: Measures local connectedness by comparing the number of connections between a node’s neighbors to the maximum possible connections.
2. Characteristic path length: Reflects global network connectivity by calculating the average number of connections between all node pairs.
3. Node degree: Represents the number of connections a node has, identifying highly connected nodes.
4. Centrality: Indicates a node’s contribution to network efficiency by its number of short-range connections.
5. Modularity: Assesses the extent of subgroup connectivity, revealing the presence of subnetworks within the larger network.

A growing range of analytical methods has been developed and applied in OSA research, advancing the understanding of the disease’s mechanisms. However, the reliability and validity of these methods still require further validation. Additionally, based on current research, we believe that different fMRI indicators hold varying clinical research potential, which should be explored in future studies.

### 3.3 Male patients with OSA versus healthy controls

Alterations in intrinsic neural networks in male patients with OSA have been investigated in 16 studies. The details of these studies are listed in Table 2, and the main findings and methods used in these studies are listed in Table 3. The methods applied in rs-fMRI studies of male patients with OSA mainly include ALFF, ReHo, ICA, seed-based FC, and graph theory.

**Table 2:** Details of the rs-fMRI studies of male patients with obstructive sleep apnea.

	Author(publication year)	No. of participants	Age, mean ± standard deviation	Apnea–hypopnea index of patient	Matched parameters between groups/Covariates
1	Zhang et al. (2013) (23)	45(24P, 21C)	P:44.6 ± 7.4 C:40.6 ± 11.4	54.7 ± 19.9	Age, years of education, and handedness
2	Peng et al. (2014) (22)	50(25P, 25C)	P:39.4 ± 1.7 C:39.5 ± 1.6	60.6 ± 18.6	Age, years of education
3	Li et al. (2015) (47)	50(25P, 25C)	P:39.4 ± 1.7 C:39.5 ± 1.6	60.0 ± 18.6	Age, years of education
4	Zhang et al. (2015) (49)	45 (24P, 21C)	P:44.6 ± 7.4 C:40.6 ± 11.4	44.6 ± 7.4	Age, years of education and handedness
5	Li et al. (2016) (57)	80 (40P, 40C)	P:38.6 ± 8.1 C:39.3 ± 7.5	59.5 ± 20.9	BMI, age and years of education
6	Li et al. (2016) (50)	80 (40P, 40C)	P:39.0 ± 8.1 C:38.8 ± 11.2	56.5 ± 19.0	Age, years of education and BMI
7	Chen et al. (2017) (55)	55 (30P, 25C)	P:38.3 ± 8.4 C:39.5 ± 8.0	62.5 ± 19.2	Age, years of education and BMI
8	Chen et al. (2018) (56)	90 (45P, 45C)	P:37.56 ± 8.86 C:37.84 ± 11.38	58.7 ± 20.38	Age, years of education and BMI
9	Chen et al. (2018) (24)	92 (46P, 46C)	20–60	58.26 ± 20.37	Age, years of education and BMI
10	Yu et al. (2019) (51)	80 (40P, 40C)	P:37.03 ± 8.74 C:38.58 ± 12.16	60.1 ± 20.45	Age, BMI
11	Kang et al. (2020) (48)	30 (14P, 16C)	P:48.71 ± 6.71 C:44.75 ± 9.26	28.89 ± 20.44	Age, BMI, years of education and altitude
12	Qin et al. (2020) (52)	74 (36P, 38C)	P:48.50 ± 7.15 C:46.13 ± 7.02	58.78 ± 21.39	Age, BMI, years of education, altitude and mean saturation of blood oxygen (MSaO2)
13	Kong et al. (2022) (53)	167 (83P, 84C)	P:40.7 ± 10.2 C:42.4 ± 12.1	51.3 ± 21.3	Age, education years, total sleep time and BMI
14	Liu et al. (2022) (54)	92 (46P, 46C)	P:38.15 ± 9.64 C:38.32 ± 11.72	27.51 ± 3.29	Age, education years, total sleep time and BMI
15	Zeng et al. (2022) (21)	114 (52P, 62C)	P:37.71 ± 9.90 C:39.69 ± 8.77	55.42 ± 21.91	Age, education years and BMI
16	Li et al. (2024) (20)	141 (69P, 72C)	P:37.56 ± 9.86 C:46.17 ± 12.49	46.17 ± 12.49	Age, BMI

No., number; P, patients; C, controls; BMI, body mass index.

**Table 3:** Resting-state fMRI studies of male patients with obstructive sleep apnea.

	Study	Methods	Results/Findings(patients vs. controls)
1	Zhang et al. (2013) (23)	ICA, VBM	FC: ↑posterior cingulate cortex (PCC) [posterior default-mode network (pDMN)]; ↓medial prefrontal cortex (MPFC) [anterior default-mode network (aDMN)], dorsolateral prefrontal cortex (DLPFC) [left frontoparietal network (LFP)], DLPFC [right frontoparietal network (RFP)]
2	Peng et al. (2014) (22)	ReHo	ReHo: ↑right posterior lobe of the cerebellum, right cin gulate gyrus (BA23), and bilateral cluster covering the lentiform nucleus, putamen, and insula (BA13) ↓right medial frontal gyrus (BA11), right superior frontal gyrus (BA10), right cluster of the precuneus and angular gyrus (BA39), and left superior parietal lobule (BA7)
3	Li et al. (2015) (47)	ALFF	ALFF: ↑left inferior frontal gyrus ↓cluster of right precuneus and bilateral posterior cingulate gyrus
4	Zhang et al. (2015) (49)	Seed-based analysis	↓FC between right AIns and the nodes of the DMN: These nodes included the MPFC, PCC, bilateral anterior cingulate cortex (ACC), bilateral superior frontal gyri (SFG), bilateral inferior parietal lobules (IPL) and right inferior temporal gyrus (ITG)
5	Li et al. (2016) (57)	Seed-based analysis	↑FC between left IPL and right IPL, and between the MPFC and left and right IPL ↓right hippocampus and the PCC, MPFC, and left MTL
6	Li et al. (2016) (50)	Voxel-wise degree centrality (DC)	DC: ↑lenticular nucleus, the putamen, posterior cerebellar ↓left middle occipital gyrus (MOG), bilateral PCC, left SFG, and bilateral IPL
7	Chen et al. (2017) (55)	Graph theory	↑normalized shortest path length, local efficiency ↓normalized clustering coefficient, small-worldness, global efficiency
8	Chen et al. (2018) (56)	Graph theory	↑characteristic path length, normalized characteristic path length ↓normalized clustering coefficient, small-worldness, global efficiency ↓nodal centralities of DMN, SN, CEN
9	Chen et al. (2018) (24)	Graph theory	↑nodal DC in the ventral medial PFC and the right parahippocampal cortex ↓clustering coefficient, local efficiency, and nodal centralities in the left PCC and DLPFC
10	Yu et al. (2019) (51)	Seed-based analysis	↑FC among left DA, anterior lobe of the cerebellum, among left VA, left IFG and left STG, between right VA and left IFG ↓FC between right DA and right PFC
11	Kang et al. (2020) (48)	ALFF, ReHo, VBM	ReHo: ↑superior frontal gyrus dorsolateral, left middle frontal gyrus, and superior frontal gyrus medial ↓left fusiform gyrus and left cerebellum lobule 6 ALFF: ↑right inferior frontal gyrus orbital part, right median cingulate and paracingulate gyri, right Inferior frontal gyrus triangular part, the right insula, left superior frontal gyrus dorsolateral
12	Qin et al. (2020) (52)	ALFF, ReHo, seed-based analysis	ReHo: ↑left superior frontal gyrus, right anterior cingulate, left parahippocampus, right postcentral gyrus, right hip pocampus and right precuneus. ↓left cuneus and the left precuneus ALFF: ↑right middle cingulate, left medial superior frontal gyrus, right anterior cingulate, right hippocampus and left parahippocampus ↓right calcarine, right inferior occipital gyrus, right middle occipital gyrus, left calcarine, right cerebellum_7b. FC: ↑ the left caudate and left thalamus
13	Kong et al. (2022) (53)	Seed-based analysis	FC: ↑bilateral SFG, right middle frontal gyrus (MFG), and left precentral gyrus (PreCG),right middle temporal gyrus(MTG) and left PreCG, right MTG and left PreCG, right inferior temporal gyrus, left lentiform nucleus, putamen and precuneus, right STG and MFG and left SFG, bilateral PreCG and right MTG, STG, and precuneus. ↓bilateral cerebellum posterior lobe (CPL) and left cerebellum anterior lobe, left CP, the bilateral CPL, fusiform gyrus, superior parietal lobule, and precuneus
14	Liu et al. (2022) (54)	Seed-based analysis	FC: ↑between the left anterior hippocampus and left middle temporal gyrus; between the left middle hippocampus and the left inferior frontal gyrus, right precentral gyrus, and left precentral gyrus; and between the left posterior hippocampus and both the right middle frontal gyrus and left middle frontal gyrus; between the right middle hippocampus and left inferior frontal gyrus; between the right middle hippocampus and left middle frontal gyrus
15	Zeng et al. (2022) (21)	ALFF	ALFF: ↑ (A) 0.01–0.1 Hz: bilateral CPL, left fusiform gyrus (FUG), left SFG, and left inferior frontal gyrus (IFG); (B) 0.198–0.25 Hz: right CPL, left MFG, left SFG, right SFG; (C) 0.073–0.198 Hz: left CPL, bilateral SFG, bilateral MFG; (D) 0.027–0.073 Hz: left CPL, right CPL, left FUG, left SFG, left IFG; (E) 0.01–0.027 Hz: right CPL, left ITG, left FUG, left IFG; (F) 0–0.01 Hz: left CPL, left IFG. ↓(A) 0.01–0.1 Hz: left MFG, bilateral PCUN/PCC, left ANG, left IPL; (B) 0.198–0.25 Hz: brainstem and bilateral PCUN/PCC; (C) 0.073–0.198 Hz: brainstem, right FUG, bilateral PCUN/PCC; (D) 0.027–0.073 Hz: bilateral PCUN/PCC, left ANG, right ANG, left IPL; (E) 0.01–0.027 Hz: bilateral PCUN/PCC.
16	Li et al. (2024) (20)	Seed-based analysis	FC: ↑the right lobule VI, right Crus I, and right Crus II

Four articles used ALFF, and three articles used ReHo. Additionally, seed-based analysis was widely employed in eight articles to measure aberrant brain synchronous activity in OSA patients. Graph theory is also increasingly utilized and is powerful for investigating the topological properties of large-scale brain networks. Furthermore, two articles used ICA to analyze aberrant brain synchronous activity in OSA patients, and only one article used voxel-wise degree centrality (DC).

ALFF and ReHo were commonly used in OSA-related rs-fMRI studies. In the study by Li et al. (2015) (47), compared with the healthy group, OSA patients showed significantly lower ALFF areas in the cluster of the right precuneus and bilateral posterior cingulate gyrus, and a higher ALFF area in the left inferior frontal gyrus. In another study by Zeng et al. (2022) (21), compared with the healthy group, the bilateral PCUN/PCC, L-ANG, R-ANG, L-IPL, R-FUG, and L-MFG ALFF values were significantly lower, while the R-cerebellum posterior lobe (CPL), L-CPL, L-fusiform gyrus (FUG), L-inferior frontal gyrus (IFG), bilateral SFG, bilateral MFG, and L-inferior temporal gyrus (ITG) ALFF values were significantly higher in the OSA group. Peng et al. (2014) (22) found that patients with OSA showed significantly lower ReHo in the right medial frontal gyrus (BA11), the right superior frontal gyrus (BA10), the right cluster of the precuneus and angular gyrus (BA39), and the left superior parietal lobule (BA7), and significantly higher ReHo in the right posterior lobe of the cerebellum, the right cingulate gyrus (BA23), and the bilateral cluster covering the lentiform nucleus, putamen, and insula (BA13). The study by Kang et al. (2020) (48) used both ALFF and ReHo. Compared with control subjects, OSA patients showed significantly increased ReHo values in the superior frontal gyrus dorsolateral, the left middle frontal gyrus, and the superior frontal gyrus medial, and significantly decreased ReHo values in the left fusiform gyrus and left cerebellum lobule 6. They also showed significantly increased ALFF values in the right inferior frontal gyrus orbital part, the right median cingulate and paracingulate gyri, the right inferior frontal gyrus triangular part, the right insula, and the left superior frontal gyrus dorsolateral.

Seed-based FC was the most commonly used method in OSA-related rs-fMRI studies. Zhang and colleagues (2015) (49) conducted a seed-based FC analysis using the anterior insula as the seed region. They examined its connectivity with key hubs of the DMN and the CEN. Compared with healthy controls, OSA patients demonstrated significantly decreased negative rsFCs between the right anterior insula (AIns) and the nodes of the DMN, and this abnormal rsFC was associated with the severity of OSA. These nodes included the MPFC, PCC, bilateral ACC, bilateral SFG, bilateral IPL, and right ITG. Significant functional disconnection was found between the right AIns and the MPFC, right SFG, left SFG, left ACC, PCC, right ITG, left IPL, and right IPL in OSA patients, which was linked with depressive scores and poorer working memory performance in OSA patients. In another study by Li et al. (2016) (50), FC between each pair of DMN sub-regions was explored in more detail. Compared with healthy controls, OSA patients displayed significantly decreased rs-FC between the right HF and the PCC, MPFC, and left MTL, and showed significantly increased rs-FC between the left IPC and right IPC, and between the MPFC and left and right IPC. Yu and colleagues (2019) (51) found that OSA patients showed significantly increased rs-FC between the left DA and the anterior lobe of the cerebellum, among the left VA, the left IFG, and the left STG, and between the right VA and the left IFG, while significantly decreased rs-FC was observed between the right DA and the right PFC. In the study by Qin et al. (2020) (52), OSA patients showed higher ReHo values in areas such as the left superior frontal gyrus, right anterior cingulate, right postcentral gyrus, left parahippocampus, right hippocampus, and right precuneus, but lower ReHo values in regions like the left cuneus and left precuneus. ALFF analysis revealed increased values in the right middle cingulate, right anterior cingulate, left parahippocampus, right hippocampus and left medial superior frontal gyrus, while decreased ALFF values were found in the right calcarine and right cerebellum. Additionally, OSA patients showed higher FC in the posterior cingulate cortex with the left caudate and thalamus. Kong and colleagues (2022) (53) found that patients with OSA showed significantly lower rs-FC with the bilateral CPL, left cerebellum anterior lobe, fusiform gyrus, superior parietal lobule, and precuneus, but significantly higher rs-FC with the bilateral SFG, right MFG, bilateral PreCG, bilateral STG, right lentiform nucleus and putamen, postcentral gyrus, right inferior temporal gyrus, and left lentiform nucleus compared to healthy controls. In the study by Liu et al. (2022), (54) in the FC analysis of the hippocampal subdivision network, compared to the healthy group, the FC in the OSA group was significantly increased between the left anterior hippocampus and left middle temporal gyrus; between the left middle hippocampus and the left inferior frontal gyrus, right precentral gyrus, and left precentral gyrus; and between the left posterior hippocampus and both the right middle frontal gyrus and left middle frontal gyrus. Similarly, the FC between the right middle hippocampus and left inferior frontal gyrus was significantly increased, as was the FC between the right middle hippocampus and left middle frontal gyrus. In the study by Li et al. (2024) (20), voxel-wise intergroup comparisons of FC across the cerebellum, based on predefined seed regions in the bilateral cerebellar lobules VI and VII, revealed distinct connectivity patterns. Specifically, the right lobule VI, right Crus I, and right Crus II showed significantly higher FC in patients with OSA compared to healthy controls.

Graph theory was occasionally utilized in rs-fMRI studies of OSA. In Chen’s 2017 study on OSA (55), graph theory was applied to rs-fMRI data. The results showed that the OSA group had significantly lower normalized clustering coefficients (*γ*), but higher normalized characteristic path lengths (*λ*) compared to healthy controls. Additionally, the small-worldness (*σ*) of the OSA group was significantly reduced. Local efficiency was notably lower in the OSA group than in the healthy group within a sparsity range of 0.09–0.15, but higher within the sparsity range of 0.23–0.40. In a study by Chen et al. (2018) (56), OSA patients showed an increase in characteristic path length (Lp) and λ, and a decrease in γ and σ, compared to healthy controls. Network efficiency was significantly lower in OSA patients. These findings indicate substantial disruption in the small-world architecture of the brain’s functional networks, along with abnormal nodal centralities in regions linked to the DMN, SN, and CEN. In another study by Chen (2018) (24), OSA patients showed decreased clustering coefficient (Cp), reduced local efficiency, and abnormal nodal centralities in the left posterior cingulate cortex and dorsal medial prefrontal cortex, while nodal centralities in the ventral medial prefrontal cortex and right parahippocampal cortex were increased. Additionally, disrupted functional connectivity within the DMN was observed, potentially contributing to topological reorganization. These findings further support the existence of cognitive deficits in OSA patients.

In the study by Zhang et al. (2013) (23), ICA was applied to rs-fMRI data, identifying seven key networks of interest. The findings revealed significantly reduced FC in OSA patients in areas such as the MPFC of the aDMN, DLPFC of the LFP and RFP, and the left precentral gyrus of the SMN. Conversely, significantly increased FC was observed only in the right PCC of the pDMN in OSA patients.

In the study by Li et al. (2016) (50), DC analysis revealed significantly reduced DC in areas such as the left middle occipital gyrus, bilateral posterior cingulate cortex, left superior frontal gyrus, and bilateral inferior parietal lobule in OSA patients compared to healthy controls. On the other hand, increased DC was found in five clusters, including the right orbital frontal cortex, bilateral cerebellum posterior lobes, and bilateral lentiform nucleus, extending to the hippocampal formation and inferior temporal gyrus.

These findings reveal that in patients with OSA, significant alterations in brain FC occur within key neural networks, such as DMN, CEN, and SN. These changes include reduced inter-network connectivity and enhanced intra-network connectivity, which may contribute to cognitive and emotional impairments. Commonly used methods such as ALFF, ReHo, seed-based analysis, graph theory, and ICA have demonstrated notable changes in regional brain activity, network synchrony, and topological properties in OSA patients. Additionally, some studies suggest that increased intra-network FC may represent a compensatory mechanism by the brain to mitigate functional deficits. These studies propose potential biomarkers based on FC alterations, offering important insights for OSA screening and diagnosis, while deepening the understanding of OSA’s impact on brain function and potential intervention strategies.

## 4 Discussion

This review analyzed 16 fMRI studies to compare brain activity differences between male patients with OSA and healthy controls. All included studies were comprehensive and met the necessary criteria. Through the descriptive analysis of this study, we found that the abnormal local brain activities, interested region related FC, and whole-brain functional connectivity networks in male patients with OSA at rest, mainly refer to brain regions in the prefrontal cortex, cerebellum, insula, basal ganglia, posterior cingulate cortex, precuneus, and angular gyrus, as well as functional networks such as the DMN, CEN, and SMN.

### 4.1 The increased-decreased local brain activities in male patients with OSA

In male OSA patients, resting-state fMRI studies based on ALFF and ReHo analyses have revealed distinct patterns of increased and decreased local brain activity (18, 34). ALFF analysis indicates that the intensity of low-frequency fluctuations is significantly increased in regions such as the left inferior frontal gyrus, bilateral superior and middle frontal gyri, the left inferior temporal gyrus, and the posterior lobe of the cerebellum, reflecting enhanced spontaneous neural activity in these areas (21, 35, 47). Similarly, ReHo analysis shows higher temporal synchronization of neural activity in the superior frontal gyrus, the left middle frontal gyrus, the right posterior cerebellum, and regions involving the lentiform nucleus, putamen, and insula, suggesting increased local neural network coordination (22, 38, 48).

In contrast, certain brain regions in OSA patients exhibit significant reductions in ALFF and ReHo. ALFF values are notably lower in the right precuneus, bilateral posterior cingulate cortex, the angular gyrus, and the left inferior parietal lobule, indicating decreased spontaneous activity in these areas (21, 47). Meanwhile, ReHo analysis reveals reduced local synchronization in the right medial and superior frontal gyri, the right precuneus, and the left fusiform gyrus, suggesting impaired neural coordination (22, 48). These alterations primarily affect brain regions related to cognition, memory, and sensory integration, highlighting the disruption of functional segregation in OSA. Such abnormal activity patterns may be closely associated with cognitive decline, attention deficits, and emotional regulation disturbances in OSA patients, providing new insights into the neurophysiological mechanisms of the disorder.

These findings indicate region-specific alterations in local neural activity in male OSA patients, with increased activity in prefrontal, cerebellar, and insular regions and decreased activity in posterior cingulate, precuneus, and parietal regions, reflecting functional impairments associated with the disease.

### 4.2 The altered FC and brain networks in male patients with OSA

In male patients with OSA, significant alterations in FC and brain networks have been observed, highlighting disruptions in key neural circuits such as the DMN, CEN, and SN. These changes manifest as reduced inter-network connectivity and enhanced intra-network connectivity, which may underlie cognitive and emotional impairments commonly associated with OSA.

Seed-based functional connectivity analysis has revealed abnormal connectivity patterns, particularly involving the anterior insula, MPFC, PCC, ACC, and other brain regions (40, 43, 49, 50). For example, decreased negative FC between the right anterior insula and DMN nodes has been linked to disease severity, depressive symptoms, and impaired working memory. Additionally, abnormal FC within the hippocampal subdivision network suggests disruptions in memory-related processes (54). Studies have also found altered connectivity in the cerebellum, fusiform gyrus, and parietal regions, further indicating widespread functional reorganization (22, 53).

ICA has provided further insights into large-scale network disruptions in OSA (23). Studies have reported decreased FC in the anterior DMN, LFP and RFP networks, and the SMN, while some regions, such as the posterior DMN, exhibited increased FC. These findings suggest that the neural connectivity alterations in OSA are not uniform but rather reflect a complex interplay of weakened and compensatory connectivity changes across different networks.

Graph theoretical analyses have demonstrated disruptions in small-world network architecture, characterized by increased characteristic path length and decreased clustering coefficients in OSA patients (18, 24, 55, 56). These changes indicate a decline in the efficiency of functional brain networks, which may contribute to cognitive deficits. Additionally, nodal centrality abnormalities in brain regions associated with the DMN, SN, and CEN suggest a reorganization of network topology, further supporting the hypothesis of OSA-induced neurofunctional impairment.

Overall, these findings indicate that male OSA patients experience widespread alterations in FC and network organization, particularly affecting regions responsible for cognitive control, memory, and emotional regulation. While some of these changes may reflect compensatory mechanisms to mitigate functional deficits, they also underscore the profound impact of OSA on brain function. These altered connectivity patterns provide important insights into the neuropathological mechanisms of OSA and may serve as potential biomarkers for diagnosis and intervention.

## 5 Conclusions and future directions

The studies discussed emphasize the significance of rs-fMRI as a promising non-invasive approach to better understanding the pathophysiology of OSA. This method offers valuable insights into brain function and connectivity, helping to identify abnormalities in OSA patients, and contributes to advancing research on the disease’s mechanisms. Rs-fMRI technology demonstrates unique advantages in the clinical application of OSA. The main advantage of rs-fMRI is its high spatial resolution, enabling precise identification of activated brain regions at the millimeter level. This provides an important imaging tool for studying the brain structure and function of OSA patients. Furthermore, rs-fMRI is easy to implement, requires minimal effort from patients, and can effectively identify functional areas in various patient groups.

Despite the potential of resting-state fMRI (rs-fMRI) in obstructive sleep apnea (OSA) research, several limitations hinder its clinical application and result consistency:

1. Lack of Standardization: Variability in data collection and preprocessing limits clinical use and multi-center collaborations.
2. Small Sample Sizes: Existing studies often have limited participants, affecting result stability and generalizability.
3. Limited Temporal Resolution: rs-fMRI measures indirect neural activity and may overlook dynamic whole-brain changes.
4. Inconsistent Correlation with OSA Severity: Discrepancies exist between rs-fMRI and rs-EEG findings, suggesting other influencing factors.
5. Challenges in Multimodal Integration: Effective fusion of EEG and fMRI data remains difficult.

To enhance the accuracy and applicability of rs-fMRI in OSA research, future efforts should focus on:

1. Standardization and Optimization: Establish unified acquisition and preprocessing protocols for clinical and multi-center use.
2. Larger and More Diverse Samples: Include broader demographics to improve reliability.
3. Multimodal Integration: Combine EEG, PET/MR for a comprehensive understanding of OSA neuropathology.
4. Advanced Analysis Methods: Utilize machine learning and graph theory for better diagnostic precision.
5. Global Collaboration and Data Sharing: Strengthen international research efforts to accelerate progress.

Future research should focus on enhancing the accuracy and practicality of rs-fMRI technology in OSA research while addressing existing technical challenges and limitations. Through these efforts, rs-fMRI is expected to become an important tool in the study and clinical diagnosis of OSA and other sleep disorders.

## Data Availability

The original contributions presented in the study are included in the article/supplementary material, further inquiries can be directed to the corresponding author/s.
